# (Acetato-κ*O*)(2-bromo-6-{[3-(dimethyl­aza­nium­yl)propyl­imino-κ*N*]meth­yl}phenolato-κ*O*)(thio­cyanato-κ*N*)zinc

**DOI:** 10.1107/S1600536812017564

**Published:** 2012-04-25

**Authors:** Cheng-Li Han

**Affiliations:** a College of Chemistry and Chemical Engineering, Qiqihar University, Qiqihar 161006, People’s Republic of China

## Abstract

In the title compound, [Zn(CH_3_COO)(NCS)(C_12_H_17_BrN_2_O)], the Zn^II^ atom is four-coordinated in a distorted tetra­hedral geometry, binding to a phenolate O and an imine N atom of the Schiff base ligand, the O atom of an acetate ligand and one thio­cyanate N atom. In the crystal, mol­ecules are linked *via* pairs of N—H⋯O hydrogen bonds, forming inversion dimers.

## Related literature

For a zinc Schiff base complex reported previously by the author, see: Han (2009 ▶). For related zinc complexes, see: Ali *et al.* (2008 ▶); You (2005 ▶); Zhu & Yang (2008 ▶).
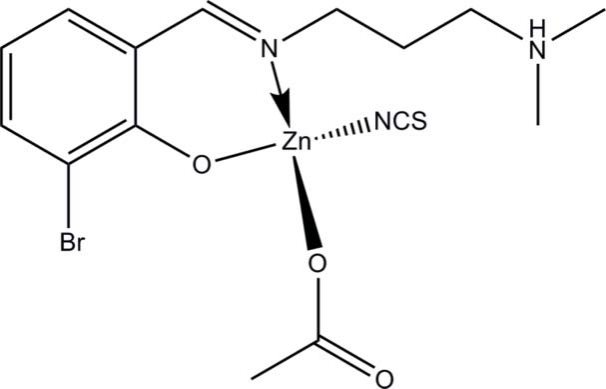


## Experimental

### 

#### Crystal data

[Zn(C_2_H_3_O_2_)(NCS)(C_12_H_17_BrN_2_O)]
*M* *_r_* = 467.68
Triclinic, 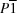

*a* = 9.3091 (6) Å
*b* = 10.2687 (6) Å
*c* = 11.8353 (7) Å
α = 66.299 (2)°
β = 79.891 (2)°
γ = 88.122 (2)°
*V* = 1018.96 (11) Å^3^
*Z* = 2
Mo *K*α radiation
μ = 3.28 mm^−1^
*T* = 298 K
0.17 × 0.15 × 0.15 mm

#### Data collection

Bruker SMART CCD area-detector diffractometer
Absorption correction: multi-scan (*SADABS*; Sheldrick, 1996 ▶) *T*_min_ = 0.605, *T*_max_ = 0.639
9684 measured reflections
3720 independent reflections
3021 reflections with *I* > 2σ(*I*)
*R*_int_ = 0.129

#### Refinement

*R*[*F* ^2^ > 2σ(*F* ^2^)] = 0.072
*wR*(*F* ^2^) = 0.201
*S* = 1.07
3720 reflections
220 parameters
H-atom parameters constrained
Δρ_max_ = 1.32 e Å^−3^
Δρ_min_ = −0.61 e Å^−3^

### 

Data collection: *SMART* (Bruker, 1998 ▶); cell refinement: *SAINT* (Bruker, 1998 ▶); data reduction: *SAINT*; program(s) used to solve structure: *SHELXS97* (Sheldrick, 2008 ▶); program(s) used to refine structure: *SHELXL97* (Sheldrick, 2008 ▶); molecular graphics: *SHELXTL* (Sheldrick, 2008 ▶); software used to prepare material for publication: *SHELXTL* and *PLATON* (Spek, 2009 ▶).

## Supplementary Material

Crystal structure: contains datablock(s) global, I. DOI: 10.1107/S1600536812017564/su2407sup1.cif

Structure factors: contains datablock(s) I. DOI: 10.1107/S1600536812017564/su2407Isup2.hkl

Additional supplementary materials:  crystallographic information; 3D view; checkCIF report

## Figures and Tables

**Table 1:** Hydrogen-bond geometry (Å, °)

*D*—H⋯*A*	*D*—H	H⋯*A*	*D*⋯*A*	*D*—H⋯*A*
N2—H2⋯O3^i^	0.91	1.81	2.703 (6)	168

Symmetry code: (i) 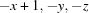.

**Fig. 1.**
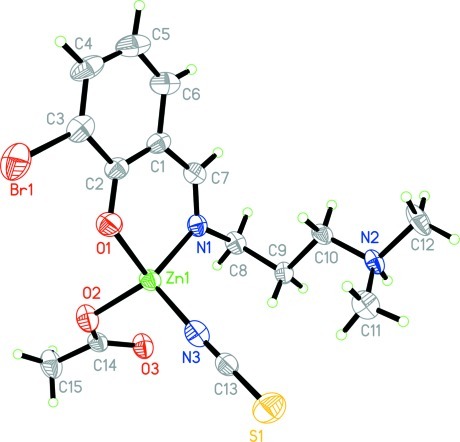
The molecular structure of the title complex, showing 30% probability displacement ellipsoids and the atom-numbering.

**Fig. 2.**
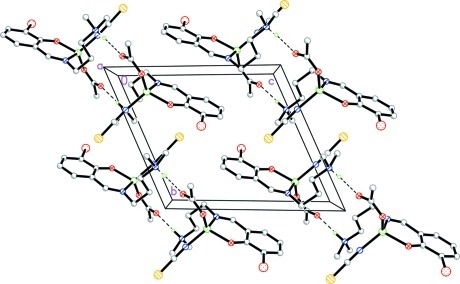
The crystal packing of the title complex viewed along the a axis, showing the formation of the inversion dimers. The N-H···O hydrogen bonds are shown as dashed lines.

## supplementary crystallographic information

### Comment

Continuing our research into the synthesis of Schiff base zinc(II) complexes (Han, 2009), we report herein on the crystal structure of the title complex. It was synthesized by the reaction of equimolar quantities of 3-bromosalicyaldehyde, N,N-dimethylpropane-1,3-diamine, ammonium thiocyanate, and zinc acetate in methanol.

In the title complex (Fig. 1), the Zn^II^ atom is four-coordinate in a tetrahedral geometry, with one O and one imine N atoms of the Schiff base ligand, one O atom of an acetate ligand, and one thiocyanate N atom. The tetrahedral geometry is severely distorted, as evidenced by the coordinate bond lengths [1.931 (4) - 1.994 (5) Å] and bond angles [96.3 (2) - 124.1 (2)°]. They are however comparable to those in similar zinc(II) complexes (Ali *et al.*, 2008; You, 2005; Zhu & Yang, 2008).

In the crystal, molecules are linked through N–H···O hydrogen bonds to form inversion dimers (Table 1 and Fig. 2).

### Experimental

Equimolar quantities (1.0 mmol each) of 3-bromosalicyaldehyde, N,N-dimethylpropane-1,3-diamine, ammonium thiocyanate, and zinc acetate were mixed in methanol. The mixture was stirred at reflux for 30 min and filtered. The filtrate was left to evaporate slowly for a few days, yielding colourless block-like crystals.

### Refinement

The NH and C-bound H-atoms were included in calculated positions and treated as riding atoms: N-H = 0.91 Å, C-H = 93, 0.97 and 0.96 Å for CH, CH_2_, and CH_3_ H-atoms, respectively, with U_iso_(H) = k × U_eq_(C), where k = 1.5 for CH_3_ H-atoms, and = 1.2 for other H-atoms. A region of disordered electron density (ca. 1.3 Å^3^) was located at position 0,0,0.5 but it could not be identified and was not taken into consideration during refinement; it corresponds to the position of a small void in the unit cell of ca. 83 Å^3^, as detected by checkcif (PLATON; Spek, 2009).

### Crystal data

**Table:**

[Zn(C_2_H_3_O_2_)(NCS)(C_12_H_17_BrN_2_O)]	*Z* = 2
*M**_r_* = 467.68	*F*(000) = 472
Triclinic, *P*1	*D*_x_ = 1.524 Mg m^−^^3^
Hall symbol: -P 1	Mo *K*α radiation, λ = 0.71073 Å
*a* = 9.3091 (6) Å	Cell parameters from 5991 reflections
*b* = 10.2687 (6) Å	θ = 2.2–27.9°
*c* = 11.8353 (7) Å	µ = 3.28 mm^−^^1^
α = 66.299 (2)°	*T* = 298 K
β = 79.891 (2)°	Block, colourless
γ = 88.122 (2)°	0.17 × 0.15 × 0.15 mm
*V* = 1018.96 (11) Å^3^	

### Data collection

**Table:**

Bruker SMART CCD area-detector diffractometer	3720 independent reflections
Radiation source: fine-focus sealed tube	3021 reflections with *I* > 2σ(*I*)
Graphite monochromator	*R*_int_ = 0.129
ω scans	θ_max_ = 25.5°, θ_min_ = 2.2°
Absorption correction: multi-scan (*SADABS*; Sheldrick, 1996)	*h* = −11→11
*T*_min_ = 0.605, *T*_max_ = 0.639	*k* = −12→12
9684 measured reflections	*l* = −14→14

### Refinement

**Table:**

Refinement on *F*^2^	Primary atom site location: structure-invariant direct methods
Least-squares matrix: full	Secondary atom site location: difference Fourier map
*R*[*F*^2^ > 2σ(*F*^2^)] = 0.072	Hydrogen site location: inferred from neighbouring sites
*wR*(*F*^2^) = 0.201	H-atom parameters constrained
*S* = 1.07	*w* = 1/[σ^2^(*F*_o_^2^) + (0.0811*P*)^2^ + 2.5468*P*] where *P* = (*F*_o_^2^ + 2*F*_c_^2^)/3
3720 reflections	(Δ/σ)_max_ < 0.001
220 parameters	Δρ_max_ = 1.32 e Å^−^^3^
0 restraints	Δρ_min_ = −0.61 e Å^−^^3^

### Special details

**Table:**

Geometry. All esds (except the esd in the dihedral angle between two l.s. planes) are estimated using the full covariance matrix. The cell esds are taken into account individually in the estimation of esds in distances, angles and torsion angles; correlations between esds in cell parameters are only used when they are defined by crystal symmetry. An approximate (isotropic) treatment of cell esds is used for estimating esds involving l.s. planes.
Refinement. Refinement of F^2^ against ALL reflections. The weighted R-factor wR and goodness of fit S are based on F^2^, conventional R-factors R are based on F, with F set to zero for negative F^2^. The threshold expression of F^2^ > 2sigma(F^2^) is used only for calculating R-factors(gt) etc. and is not relevant to the choice of reflections for refinement. R-factors based on F^2^ are statistically about twice as large as those based on F, and R- factors based on ALL data will be even larger.

### Fractional atomic coordinates and isotropic or equivalent isotropic displacement parameters (Å^2^)

**Table:**

	*x*	*y*	*z*	*U*_iso_*/*U*_eq_	
Zn1	0.71920 (7)	0.16314 (7)	0.17582 (7)	0.0445 (3)	
Br1	0.86805 (11)	0.41595 (10)	0.41002 (10)	0.0872 (4)	
N1	0.5145 (5)	0.0900 (5)	0.2551 (5)	0.0429 (11)	
N2	0.2396 (5)	0.2871 (5)	−0.0322 (5)	0.0436 (11)	
H2	0.2172	0.2258	−0.0653	0.052*	
N3	0.7308 (6)	0.2974 (7)	0.0016 (6)	0.0642 (15)	
O1	0.7416 (5)	0.2599 (5)	0.2823 (5)	0.0599 (12)	
O2	0.8893 (5)	0.0442 (5)	0.1972 (5)	0.0598 (12)	
O3	0.7888 (5)	−0.0922 (5)	0.1276 (5)	0.0565 (11)	
S1	0.7396 (3)	0.5055 (2)	−0.2352 (2)	0.0807 (6)	
C1	0.5007 (7)	0.1983 (6)	0.4068 (5)	0.0478 (14)	
C2	0.6446 (7)	0.2621 (6)	0.3737 (6)	0.0475 (14)	
C3	0.6772 (9)	0.3331 (6)	0.4473 (7)	0.0589 (17)	
C4	0.5794 (11)	0.3442 (7)	0.5422 (7)	0.070 (2)	
H4	0.6069	0.3901	0.5891	0.084*	
C5	0.4342 (11)	0.2853 (8)	0.5701 (7)	0.075 (2)	
H5	0.3646	0.2963	0.6320	0.090*	
C6	0.4007 (9)	0.2134 (7)	0.5044 (6)	0.0640 (19)	
H6	0.3073	0.1718	0.5243	0.077*	
C7	0.4463 (7)	0.1182 (6)	0.3462 (6)	0.0473 (14)	
H7	0.3503	0.0826	0.3767	0.057*	
C8	0.4325 (7)	0.0049 (6)	0.2105 (6)	0.0486 (14)	
H8A	0.3428	−0.0341	0.2699	0.058*	
H8B	0.4903	−0.0740	0.2065	0.058*	
C9	0.3960 (6)	0.0940 (6)	0.0817 (6)	0.0430 (13)	
H9A	0.4839	0.1438	0.0248	0.052*	
H9B	0.3587	0.0321	0.0485	0.052*	
C10	0.2827 (6)	0.2011 (7)	0.0899 (6)	0.0468 (14)	
H10A	0.1967	0.1508	0.1495	0.056*	
H10B	0.3217	0.2641	0.1210	0.056*	
C11	0.3592 (8)	0.3865 (8)	−0.1235 (8)	0.0659 (19)	
H11A	0.3239	0.4437	−0.1991	0.099*	
H11B	0.4392	0.3329	−0.1423	0.099*	
H11C	0.3916	0.4470	−0.0879	0.099*	
C12	0.1071 (7)	0.3664 (8)	−0.0167 (9)	0.074 (2)	
H12A	0.0287	0.3006	0.0379	0.110*	
H12B	0.0797	0.4187	−0.0969	0.110*	
H12C	0.1269	0.4314	0.0192	0.110*	
C13	0.7343 (6)	0.3843 (7)	−0.0990 (6)	0.0471 (14)	
C14	0.8872 (6)	−0.0647 (6)	0.1725 (6)	0.0418 (13)	
C15	1.0122 (8)	−0.1603 (8)	0.2014 (8)	0.068 (2)	
H15A	0.9755	−0.2559	0.2535	0.102*	
H15B	1.0720	−0.1286	0.2445	0.102*	
H15C	1.0694	−0.1579	0.1247	0.102*	

### Atomic displacement parameters (Å^2^)

**Table:**

	*U*^11^	*U*^22^	*U*^33^	*U*^12^	*U*^13^	*U*^23^
Zn1	0.0424 (4)	0.0461 (4)	0.0530 (5)	0.0063 (3)	−0.0081 (3)	−0.0288 (3)
Br1	0.1033 (7)	0.0773 (6)	0.1037 (7)	−0.0066 (5)	−0.0388 (6)	−0.0506 (5)
N1	0.041 (2)	0.038 (2)	0.050 (3)	0.0034 (19)	−0.012 (2)	−0.017 (2)
N2	0.036 (2)	0.036 (2)	0.073 (3)	0.0091 (18)	−0.018 (2)	−0.034 (2)
N3	0.047 (3)	0.075 (4)	0.072 (4)	0.007 (3)	−0.010 (3)	−0.031 (3)
O1	0.056 (2)	0.069 (3)	0.072 (3)	−0.001 (2)	−0.008 (2)	−0.047 (3)
O2	0.053 (2)	0.057 (3)	0.095 (4)	0.016 (2)	−0.027 (2)	−0.052 (3)
O3	0.052 (2)	0.055 (2)	0.082 (3)	0.0154 (19)	−0.024 (2)	−0.044 (2)
S1	0.0938 (15)	0.0725 (13)	0.0613 (12)	−0.0063 (11)	−0.0086 (11)	−0.0134 (10)
C1	0.067 (4)	0.038 (3)	0.039 (3)	0.013 (3)	−0.012 (3)	−0.017 (2)
C2	0.062 (4)	0.039 (3)	0.049 (3)	0.013 (3)	−0.014 (3)	−0.024 (3)
C3	0.088 (5)	0.037 (3)	0.057 (4)	0.013 (3)	−0.023 (4)	−0.020 (3)
C4	0.127 (7)	0.044 (3)	0.049 (4)	0.018 (4)	−0.021 (4)	−0.027 (3)
C5	0.112 (7)	0.058 (4)	0.051 (4)	0.016 (4)	0.001 (4)	−0.025 (3)
C6	0.084 (5)	0.047 (3)	0.051 (4)	0.011 (3)	0.001 (3)	−0.015 (3)
C7	0.047 (3)	0.040 (3)	0.048 (3)	0.002 (2)	−0.004 (3)	−0.012 (3)
C8	0.046 (3)	0.040 (3)	0.062 (4)	0.003 (2)	−0.011 (3)	−0.022 (3)
C9	0.046 (3)	0.041 (3)	0.057 (3)	0.012 (2)	−0.020 (3)	−0.032 (3)
C10	0.042 (3)	0.051 (3)	0.061 (4)	0.012 (2)	−0.010 (3)	−0.035 (3)
C11	0.061 (4)	0.051 (4)	0.079 (5)	−0.003 (3)	−0.008 (4)	−0.021 (4)
C12	0.048 (3)	0.063 (4)	0.130 (7)	0.027 (3)	−0.028 (4)	−0.057 (5)
C13	0.038 (3)	0.053 (3)	0.055 (4)	0.002 (2)	−0.005 (3)	−0.027 (3)
C14	0.040 (3)	0.040 (3)	0.052 (3)	0.010 (2)	−0.007 (2)	−0.026 (3)
C15	0.058 (4)	0.059 (4)	0.104 (6)	0.023 (3)	−0.031 (4)	−0.045 (4)

### Geometric parameters (Å, º)

**Table:**

Zn1—O1	1.931 (4)	C5—C6	1.345 (11)
Zn1—N3	1.952 (7)	C5—H5	0.9300
Zn1—O2	1.957 (4)	C6—H6	0.9300
Zn1—N1	1.994 (5)	C7—H7	0.9300
Br1—C3	1.897 (8)	C8—C9	1.523 (9)
N1—C7	1.284 (8)	C8—H8A	0.9700
N1—C8	1.476 (8)	C8—H8B	0.9700
N2—C10	1.477 (8)	C9—C10	1.518 (7)
N2—C12	1.482 (7)	C9—H9A	0.9700
N2—C11	1.488 (8)	C9—H9B	0.9700
N2—H2	0.9100	C10—H10A	0.9700
N3—C13	1.161 (9)	C10—H10B	0.9700
O1—C2	1.289 (7)	C11—H11A	0.9600
O2—C14	1.265 (7)	C11—H11B	0.9600
O3—C14	1.229 (7)	C11—H11C	0.9600
S1—C13	1.586 (7)	C12—H12A	0.9600
C1—C6	1.407 (9)	C12—H12B	0.9600
C1—C2	1.430 (9)	C12—H12C	0.9600
C1—C7	1.442 (9)	C14—C15	1.493 (8)
C2—C3	1.414 (9)	C15—H15A	0.9600
C3—C4	1.356 (11)	C15—H15B	0.9600
C4—C5	1.428 (13)	C15—H15C	0.9600
C4—H4	0.9300		
			
O1—Zn1—N3	111.3 (2)	N1—C8—H8A	109.3
O1—Zn1—O2	100.4 (2)	C9—C8—H8A	109.3
N3—Zn1—O2	111.2 (2)	N1—C8—H8B	109.3
O1—Zn1—N1	96.3 (2)	C9—C8—H8B	109.3
N3—Zn1—N1	111.3 (2)	H8A—C8—H8B	108.0
O2—Zn1—N1	124.1 (2)	C10—C9—C8	110.5 (5)
C7—N1—C8	116.7 (5)	C10—C9—H9A	109.5
C7—N1—Zn1	121.0 (4)	C8—C9—H9A	109.5
C8—N1—Zn1	122.2 (4)	C10—C9—H9B	109.5
C10—N2—C12	111.3 (5)	C8—C9—H9B	109.5
C10—N2—C11	112.8 (5)	H9A—C9—H9B	108.1
C12—N2—C11	110.2 (5)	N2—C10—C9	112.7 (5)
C10—N2—H2	107.4	N2—C10—H10A	109.1
C12—N2—H2	107.4	C9—C10—H10A	109.1
C11—N2—H2	107.4	N2—C10—H10B	109.1
C13—N3—Zn1	175.3 (6)	C9—C10—H10B	109.1
C2—O1—Zn1	125.7 (4)	H10A—C10—H10B	107.8
C14—O2—Zn1	117.7 (4)	N2—C11—H11A	109.5
C6—C1—C2	119.7 (6)	N2—C11—H11B	109.5
C6—C1—C7	115.5 (6)	H11A—C11—H11B	109.5
C2—C1—C7	124.8 (5)	N2—C11—H11C	109.5
O1—C2—C3	119.9 (6)	H11A—C11—H11C	109.5
O1—C2—C1	124.3 (5)	H11B—C11—H11C	109.5
C3—C2—C1	115.8 (6)	N2—C12—H12A	109.5
C4—C3—C2	123.3 (7)	N2—C12—H12B	109.5
C4—C3—Br1	119.1 (6)	H12A—C12—H12B	109.5
C2—C3—Br1	117.6 (5)	N2—C12—H12C	109.5
C3—C4—C5	120.0 (7)	H12A—C12—H12C	109.5
C3—C4—H4	120.0	H12B—C12—H12C	109.5
C5—C4—H4	120.0	N3—C13—S1	178.7 (6)
C6—C5—C4	118.3 (7)	O3—C14—O2	122.9 (5)
C6—C5—H5	120.9	O3—C14—C15	120.9 (5)
C4—C5—H5	120.9	O2—C14—C15	116.2 (6)
C5—C6—C1	122.9 (8)	C14—C15—H15A	109.5
C5—C6—H6	118.6	C14—C15—H15B	109.5
C1—C6—H6	118.6	H15A—C15—H15B	109.5
N1—C7—C1	127.8 (6)	C14—C15—H15C	109.5
N1—C7—H7	116.1	H15A—C15—H15C	109.5
C1—C7—H7	116.1	H15B—C15—H15C	109.5
N1—C8—C9	111.6 (5)		

### Hydrogen-bond geometry (Å, º)

**Table:**

*D*—H···*A*	*D*—H	H···*A*	*D*···*A*	*D*—H···*A*
N2—H2···O3^i^	0.91	1.81	2.703 (6)	168

Symmetry code: (i) −*x*+1, −*y*, −*z*.

## References

[bb1] Ali, H. M., Mohamed Mustafa, M. I., Rizal, M. R. & Ng, S. W. (2008). *Acta Cryst.* E**64**, m718–m719. 10.1107/S1600536808011161 PMC2961161 21202245

[bb2] Bruker (1998). *SMART* and *SAINT* Bruker AXS Inc., Madison, Wisconsin, USA.

[bb3] Han, C.-L. (2009). *Acta Cryst.* E**65**, m418. 10.1107/S1600536809009234 PMC2968908 21582360

[bb4] Sheldrick, G. M. (1996). *SADABS* University of Göttingen, Germany.

[bb5] Sheldrick, G. M. (2008). *Acta Cryst.* A**64**, 112–122. 10.1107/S0108767307043930 18156677

[bb6] Spek, A. L. (2009). *Acta Cryst.* D**65**, 148–155. 10.1107/S090744490804362X PMC2631630 19171970

[bb7] You, Z.-L. (2005). *Acta Cryst.* E**61**, m1571–m1573.

[bb8] Zhu, X.-W. & Yang, X.-Z. (2008). *Acta Cryst.* E**64**, m1090–m1091. 10.1107/S1600536808023659 PMC2961998 21203068

